# Intrahepatic Biliary Colloid Carcinoma: A Rare Site for Recurrence

**DOI:** 10.7759/cureus.41925

**Published:** 2023-07-15

**Authors:** Luis M Nieto, John Martinez, Sharon I Narvaez, Steven Keilin

**Affiliations:** 1 Internal Medicine, Wellstar Cobb Hospital, Austell, USA; 2 Escuela de Medicina, Universidad de Guayaquil, Guayaquil, ECU; 3 Pancreaticobiliary Service, Division of Gastroenterology, Emory School of Medicine, Atlanta, USA

**Keywords:** jaundice, recurrence, biliary duct, pancreas, colloid carcinoma

## Abstract

A new biliary duct (BD) stricture raises questions about the presence of malignancy, especially with a history of metastatic pancreatic cancer. A few cases of colloid carcinoma (CC) of the pancreas have been published, but none have described recurrence in the biliary tract. We report a case of intrahepatic biliary CC that recurred after two years after the last dose of immunotherapy for pancreatic CC. In addition to a unique biliary cancer case presentation, this case raises awareness of the best strategy for cancer surveillance.

## Introduction

Colloid carcinoma (CC) of the pancreas is a rare variant of ductal adenocarcinoma (DAC) and represents 1% of pancreatic carcinomas [1]. Most CC cases develop from intrapapillary mucinous neoplasm (IPMN) [2]. CC is also recognized as a histological subtype of invasive IPMN, and its differentiation from IPMN can be challenging. CC has been shown to have better overall survival than DAC [3], but its recurrence rate has yet to be established. Also, the liver is the most common site of distant metastases in invasive pancreatic IPMN, but its frequency has not been described in CC metastases [4]. To our knowledge, no other case of pancreatic CC with multiple liver metastatic lesions and intrahepatic biliary duct (BD) recurrence has been reported in English literature.

This article was previously presented as a meeting abstract at the ACG Annual Scientific Meeting on October 25, 2020.

## Case presentation

**Figure 1:**
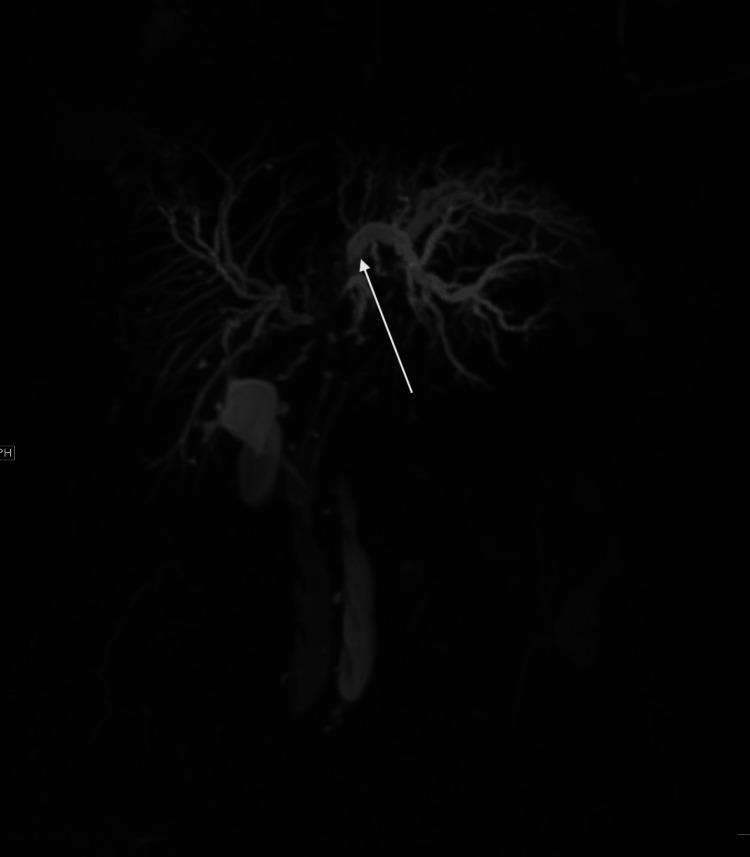
MRCP showing left intrahepatic BD dilation. MRCP: Magnetic resonance cholangiopancreatography; BD: Biliary duct.

A 72-year-old female with a history of stage IV pancreatic CC with metastases to the liver, spleen, and stomach presented with dark urine and jaundice. Diagnosed five years earlier, she required distal pancreatectomy, splenectomy, and partial gastrectomy. At that time, pathology showed poorly differentiated CC of the pancreas, invading the spleen and stomach with negative surgical margins. Unfortunately, one month later, metastatic liver lesions were found during post-surgical follow-up. She failed gemcitabine, paclitaxel, liver CT-guided microwave ablation, and a combination of leucovorin calcium, fluorouracil, and oxaliplatin therapy. Pembrolizumab was started for progression and showed stable disease for two years but was stopped due to neutropenia. Two years after the last dose of immunotherapy, magnetic resonance cholangiopancreatography (MRCP) for surveillance showed new moderate left intrahepatic duct dilation with left hepatic duct narrowing (Figure 1). 

**Figure 2:**
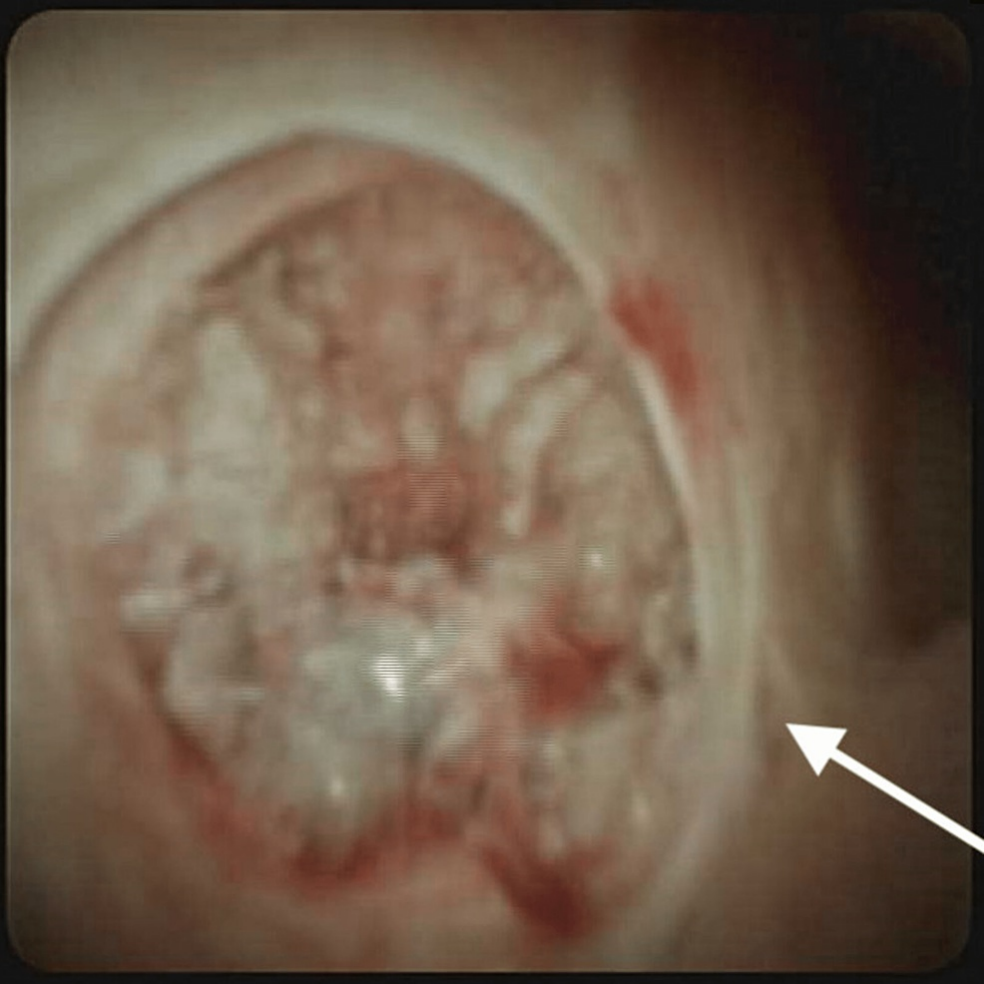
Frondlike mass in the left hepatic main duct.

**Figure 3:**
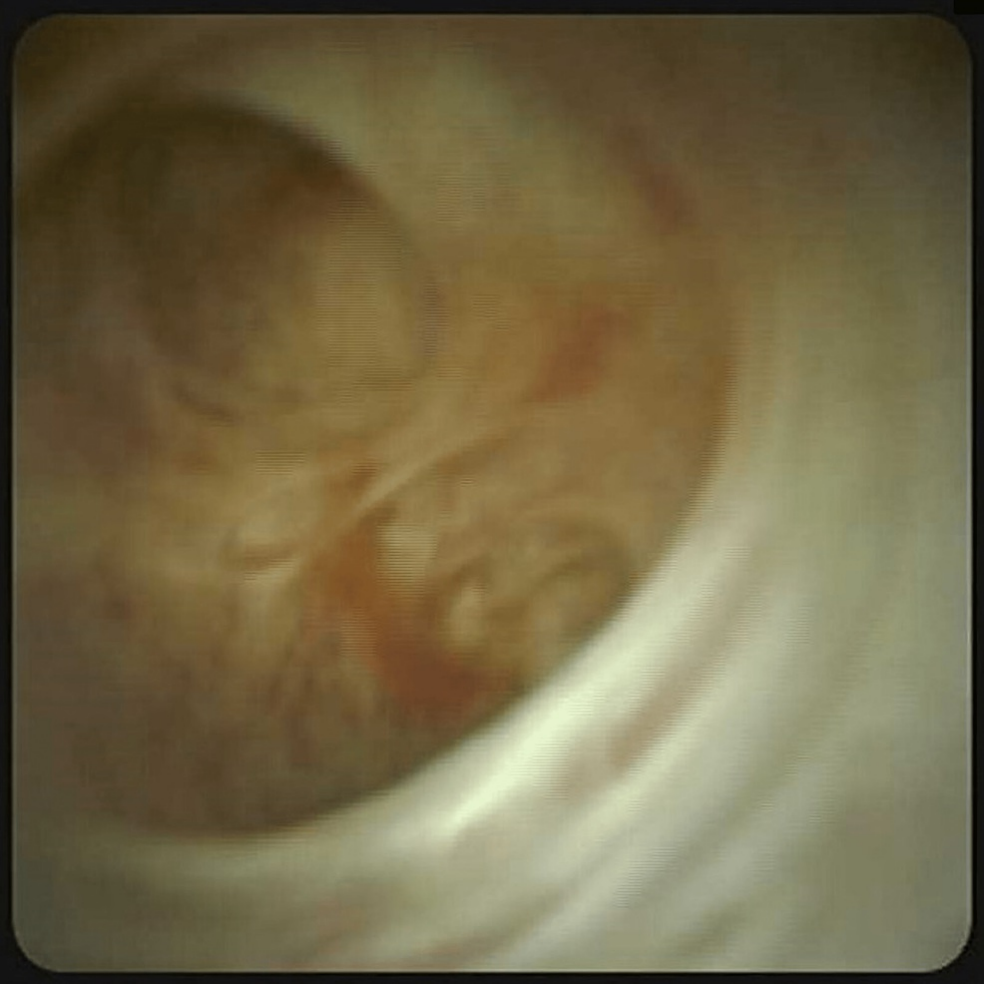
Normal right hepatic main duct under cholangioscopy.

She underwent endoscopic retrograde cholangiopancreatography (ERCP) twice, requiring biliary stent placement and brush cytology with benign results. Discussion at the multidisciplinary tumor board led to a consensus on the intraductal papillary mucinous neoplasm of the bile duct (IPMN-B). During the most recent hospitalization, labs showed elevated liver function studies with a total bilirubin of 4 mg/dl. ERCP was done with cholangioscopy, demonstrating a large frond-like mass partially obstructing the left main hepatic duct (Figures 2-3). 

It was biopsied, and pathology was reported as CC.

## Discussion

CC of the pancreas is a sub-category of DAC differentiated by histopathology, and at least half of the epithelial cells are mucinous components comprising the tumor [5]. The mucin surrounding the neoplastic cell serves as a barrier preventing further tumor invasion. Mucin gene 1 (MUC1) is a surface glycoprotein strongly expressed only in DAC. Mucin gene 2 (MUC2) is a surface glycoprotein with tumor suppressor activity commonly expressed in CC and IPMN but not in DAC [6]. It is believed that MUC2 is more microsatellite stable than MUC1. In addition, MUC 2 usually does not exhibit a mutation in the mismatch repair genes [3,7]. Microsatellite stability on MUC2 is one of the reasons why the clinical presentation of CC is more favorable than DAC. Our patient demonstrated this, as she remained stable for two years since the last medical therapy.

Clinical presentations in CC of the pancreas are often non-specific. Patients can present with nausea, vomiting, abdominal pain, back pain, weight loss, or anorexia. In cases where the tumor obstructs the main pancreatic duct, the patient can experience pancreatic insufficiency and malabsorption of nutrients [7]. However, a significant number of patients remain asymptomatic, and the detection of CC is often incidental. Distinguishing between ductal adenocarcinoma (DAC), CC, and intraductal papillary mucinous neoplasm (IPMN) can be challenging due to their close resemblance [5]. The laboratory data can show elevated serum of cancer antigen 19-9 (CA19-9) and carcinoembryonic antigen (CEA), and they could have a similar presentation on imaging [8,9]. However, their histology and MUC gene expression are what differentiate them. In our case, the patient had CC of the pancreas for five years. After this period, the neoplasm metastasized to the left intrahepatic duct, posing a challenge in distinguishing between CC of the bile duct and intraductal papillary mucinous neoplasm of the bile duct (IPMN-B) due to their similar histology and MUC gene expression. Both entities can share common signs, most notably jaundice and dark urine. While many patients with CC remain asymptomatic, some may experience right upper quadrant abdominal pain, nausea, vomiting, and weight loss. Our patient, however, presented solely with jaundice and dark urine. We have seen data showing the common sites of metastasis from IPMN, making the invasion of the bile duct and pancreas the most common site, followed by liver, renal, intestinal, and gastric [7]. To our understanding, no data on CC metastasis site and recurrence has been reported in English literature.

The diagnostic approach typically begins with imaging, where MRI and CT scans are less invasive and highly useful prior to surgery. MRCP can be helpful to recognize BD strictures and dilation of the ducts. As in our case, the presence of a new stricture raises concerns about malignancy. Performing ERCP with cholangioscopy can be particularly helpful in identifying lesions and obtaining more accurate pathology samples. A frond-like mass on BD under cholangioscopy raises suspicion for malignancy and warrants a direct lesion biopsy. Endoscopic ultrasound with a fine-needle aspiration can also aid in confirming malignancy [10,11]. It is challenging to differentiate between IPMN-B and CC of the bile ducts due to the similarity in the histopathology. However, certain distinctions can aid in differentiation, such as hypersecretion into the ductal lumen and excessive mucin in IPMN-B [12]. our patient's case demonstrates CC of the bile duct, that makes it unique from IPMN and IPMN-B. The type of cells found in CC tends to store the mucin inside the cell without any secretion, resulting in intracellular mucin deposition with peripheral displacement of the nucleus on histology [2, 13]. Currently, management for CC of the pancreas remains non-specific. The first-line therapy typically involves a surgical approach followed by a chemotherapy regimen, with remission achieved in less than 20% of cases. Additionally, a small percentage may experience recurrence and metastasis [14].

## Conclusions

In conclusion, our case highlights the challenges in distinguishing between IPMN-B and CC of the BD. A new BD stricture raised concerns about the presence of malignancy, especially given the patient's history of metastatic pancreatic cancer. The cholangioscopy findings and the pathology report should make us investigate the best strategy for cancer surveillance since brush cytology can often yield negative results. Further studies are warranted to explore the recurrence and metastasis rates in pancreatic CC.
